# Erasmus+sport let’s move Europa: learning units for health promotion among children and adolescents

**DOI:** 10.1093/eurpub/ckac131.463

**Published:** 2022-10-25

**Authors:** A Masini, G Zanutto, G Longo, S Marini, G Soldà, A Salussolia, A Anastasia, D Sánchez-Oliva, A Ceciliani, L Dallolio

**Affiliations:** 1 Department of Biomedical and Neuromotor Sciences, University of Bologna, Bologna, Italy; 2 Department for Life Quality Studies, University of Bologna, Rimini, Italy; 3 Faculty of Sport Sciences, University of Extremadura, Cáceres, Spain

## Abstract

**Background:**

School years are crucial for acquiring life-lasting healthy habits. However, an increasing rate of children and adolescents fail to maintain a healthy lifestyle. European Union has financed the Erasmus+ Sport Let’s Move Europa project to design an innovative digital tool for promoting healthy lifestyles among those age groups. University of Bologna, partner of the project, has developed 30 Learning Units (LUs) about Physical Activity, Sleep and Nutrition to be integrated in the school program by teachers.

**Methods:**

A group of 17 teachers (15 females and 2 males, mean SD age 49,5 ±11,6) from primary and secondary schools located in Bologna province (Italy) took part in semi-structured focus groups (FGs). The investigation focused on facilitators/barriers of the intervention and possible solutions, identifying time frames and locations, suggestions for engaging the different stakeholders (teachers, students and families). All FGs were recorded, transcribed, anonymized, and analyzed through inductive thematic analysis.

**Results:**

30 LUs were created based on the latest scientific evidence and the FGs output. Each LU addresses a specific topic and is tailored differently for primary and secondary school. The layout includes an investigation on the topic, classroom activities, and a section about “healthy homework” or “challenges” to be accomplished at home, engaging families in the construction of a healthy routine. All the activities were designed to be feasible and sustainable. Each LU includes a discussion phase to understand students’ feedback about proposed homework and learning content.

**Conclusions:**

FGs have proven crucial to tailoring LUs on the needs of different stakeholders and co-designing an effective intervention. “Healthy homework” and “Challenges” encourage students to pursue healthy habits also outside the school setting, involving families. Feedback on the activity provides an insight into the progression and effectiveness of the intervention.

**Key messages:**

• The EUmove project integrates knowledge about sleeping, nutritional and physical activity habits into the school curriculum to promote healthy lifestyles among students and their families.

• Thanks to the FGs, LUs are designed to suit the target audience. LUs integration into school curricula is therefore a feasible intervention, not requiring specialized personnel to be implemented.

